# Cartilage Acidic Protein a Novel Therapeutic Factor to Improve Skin Damage Repair?

**DOI:** 10.3390/md19100541

**Published:** 2021-09-25

**Authors:** Rute Castelo Félix, Liliana Anjos, Rita Alves Costa, Sophia Letsiou, Deborah Mary Power

**Affiliations:** 1 Centro de Ciências do Mar (CCMAR), Comparative Endocrinology and Integrative Biology Group, Universidade do Algarve, Campus de Gambelas, 8005-139 Faro, Portugal; lanjos@ualg.pt (L.A.); racosta@ualg.pt (R.A.C.); 2 Laboratory of Biochemistry, Scientific Affairs, APIVITA SA, Industrial Park of Markopoulo Mesogaias, Markopoulo Attikis, 19003 Athens, Greece; letsiou-s@apivita.com; 3 International Research Center for Marine Biosciences, Ministry of Science and Technology, Shanghai Ocean University, Shanghai 201306, China; 4 Key Laboratory of Exploration and Utilization of Aquatic Genetic Resources, Ministry of Education, Shanghai Ocean University, Shanghai 201306, China

**Keywords:** electric cell impedance sensing (ECIS), fish skin fibroblast, scratch assay, vertebrate CRTAC1, zebrafish

## Abstract

Fish skin has been gaining attention due to its efficacy as a human-wound-treatment product and to identify factors promoting its enhanced action. Skin fibroblasts have a central role in maintaining skin integrity and secrete extra cellular matrix (ECM) proteins, growth factors and cytokines to rapidly repair lesions and prevent further damage or infection. The effects on scratch repair of the ubiquitous but poorly characterized ECM protein, cartilage acidic protein 1 (CRTAC1), from piscine and human sources were compared using a zebrafish SJD.1 primary fibroblast cell line. A classic in vitro cell scratch assay, immunofluorescence, biosensor and gene expression analysis were used. Our results demonstrated that the duplicate sea bass Crtac1a and Crtac1b proteins and human CRTAC-1A all promoted SJD.1 primary fibroblast migration in a classic scratch assay and in an electric cell impedance sensing assay. The immunofluorescence analysis revealed that CRTAC1 enhanced cell migration was most likely caused by actin-driven cytoskeletal changes and the cellular transcriptional response was most affected in the early stage (6 h) of scratch repair. In summary, our results suggest that CRTAC1 may be an important factor in fish skin promoting damage repair.

## 1. Introduction

The skin is the largest organ in the body and functions as a protective barrier, a thermoregulatory and sensory organ and produces multi-functional molecules such as hormones and enzymes [1,2]. Constant exposure of the skin to the environment due to normal “wear and tear” can lead to loss of structural integrity, and regeneration or healing to restore integrity is of utmost importance [2]. Skin wound healing in vertebrates is complex and involves multiple phases including, inflammation, proliferation and remodeling. Resident and non-resident cell types are involved in repair, and the resident dermal fibroblasts play a central role in the secretion of the extracellular matrix (ECM), growth factors and cytokines needed for repair [3,4,5,6,7,8,9]. The malfunctioning of fibroblasts in a diversity of tissues can lead to severe disease due to diminished or excess ECM deposition leading to progressive tissue scarring and even organ dysfunction (e.g., liver cirrhosis; kidney fibrosis and cardiac fibrosis) [7,10,11]. For example, reduced capacity of fibroblasts to sustain proliferation and tissue repair contribute to the evolution of pulmonary emphysema [12,13]. In skin, dysregulation of fibroblast function causes fibrosis, hypertrophic scars or keloids [7]. 

Non-structural matricellular proteins of the ECM such as galectins, osteopontin, SPARC (a.k.a. osteonectin) and tenascins trigger cell-specific activities [14,15] and are up-regulated at sites of tissue remodeling in vertebrates [15,16]. Cartilage acidic protein 1 (CRTAC1) a calcium-binding ECM protein with a widespread tissue distribution, has been proposed as a biomarker and a potential therapeutic target for diseases of the human cardiovascular, respiratory and urinary systems [17] and protects against UVB induced apoptosis of human epithelial cells [18,19]. In humans two CRTAC1 splice variants have been described: a long form designated CRTAC1-A and a short form designated CRTAC1-B [20]. In teleost fish, due to the teleost specific whole genome duplication (TWGD, [21]), gene duplicates, Crtac1a and Crtac1b, that share higher amino acid sequence conservation with human CRTAC1-A exist [22]. The presence of an N-terminal integrin-a chain-like domain and a C-terminal EGF-like Ca-binding motif in CRTAC1 proteins reinforces their likely importance in cell–matrix interactions. CRTAC1 was recently shown to promote migration of normal human primary dermal fibroblasts [23] and fish skin primary epithelial cells in vitro [24].

The zebrafish (*Danio rerio*) SJD.1 primary fibroblast cell line used in the present study was isolated from amputated caudal fins from adult zebrafish and retains many features of non-transformed cells such as eudiploidy, contact inhibition, and surface adhesion [25]. The SJD.1 cell line has been used as a model to study in vitro viral infections in fish [26] and mechanisms of differential regulation of genes by metal ion toxicity [27]. The stimulation by fish skin or fish collagen and other ECM proteins of human skin regeneration and recovery after burns has heightened interest in the characteristics of wound healing in fish [28,29,30]. Moreover, with an increase in the elderly population and rise in age-related pathologies (e.g., diabetes, etc.) chronic non-healing wounds have become a major medical challenge [31]. This has led to the development of cellular and tissue-based therapies (CTPs) for the treatment of chronic wounds and the success of fish skin xenografts has raised interest in identifying mediators of the effect [32,33]. Furthermore, explaining why fish skin neither scars or wrinkles and has greater regenerative capacity than mammalian skin is of great interest [34] particularly because the main steps of skin regeneration in vivo are shared [35,36,37,38,39]. 

In the present study with a view to characterizing and identifying the unique characteristics of fish skin fibroblasts, skin primary fibroblasts, SJD.1, from zebrafish were evaluated and the response to the abundant skin ECM protein, CRTAC1, was assessed using an in vitro scratch recovery model. The relative activity and mechanisms of action of human CRTAC1-A (hCRTAC1-A) was compared to the duplicate recombinant proteins, dlCrtac1a and dlCrtac1b, from seabass (*Dicentrarchus labrax*). Classic in vitro cell scratch assays (scratch assay), immunofluorescence assays (IFA), biosensor assays (electric cell impedance system (ECIS)) and gene expression analysis (RT-qPCR assays) were used. The expression of homologues of mammalian genes involved in wound healing in mammals (cell proliferation, apoptosis, extracellular matrix, antioxidant, differentiation, and migration), was characterized during scratch repair and the effect of the ECM protein CRTAC1 from human and fish was compared. Our results revealed that, although CRTAC1 proteins from human and fish slightly differ in their capacity to stimulate scratch repair by zebrafish SJD.1 fibroblasts, they all significantly promoted fibroblast migration in the scratch assay.

## 2. Results

### 2.1. SJD.1 Cell Culture

Zebrafish SJD.1 primary fibroblasts are adherent cells. Preliminary studies revealed that they proliferate slowly with a doubling time of five days (Figure S1). Immunofluorescence analysis (IF) revealed that the cells have a prominent centrally located nuclei and a well-developed cytoskeleton with abundantly labelled actin (red) and tubulin (green) filaments (Figure S2).

### 2.2. Classic Scratch Assay

**Figure 1:**
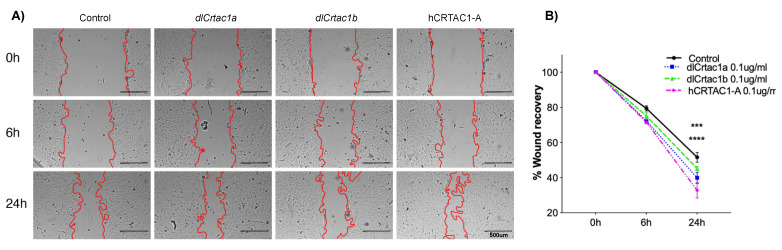
SJD.1 fibroblast scratch assay: (**A**) Representative images showing the progression of the scratch closure across time in control and cartilage acidic protein 1 (CRTAC1) (dlcrtac1a, dlcrtac1b and hCRTAC1-A) exposed fibroblasts. The scratch repaired more rapidly in the CRTAC1 exposed cells. Photos were taken with a Leica DM IL microscope coupled to a Visicam HDMI 6 digital camera (magnification × 4). Scale bars indicate 500 μm; and (**B**) scratch recovery area (percentage) was measured at 0 h, 6 h and 24 h in relation to the area immediately after the scratch (100%). The results are shown as the average ± SEM of five independent experiments with two technical replicates. The data were analyzed using a two-way ANOVA followed by Tukey’s Multiple Comparison test. The statistical analysis was performed using GraphPad Prism version 7.0a. *p* < 0.0001 (****) and *p* < 0.0005 (***) were considered significant.

Scratch repair assays were performed with SJD.1 primary fibroblasts and monitored at 6 and 24 h after the scratch (*n* = 5 independent experiments with two technical replicates). For the control and vertebrate CRTAC1 treatment groups, approximately 60–70% of scratch recovery (expressed as % of the total scratch area at time 0) was achieved 24 h after scratching in the control and all treatments (Figure 1A). The scratch repair was faster in cells exposed to CRTAC1 compared to the untreated control. However, only hCRTAC1-A and dlCrtac1a caused a highly significant enhancement (*p* < 0.001 and *p* < 0.005, respectively) in scratch recovery compared to the control cells (Figure 1B).

### 2.3. Electrical Cell Impedance System (ECIS) Analysis

**Figure 2:**
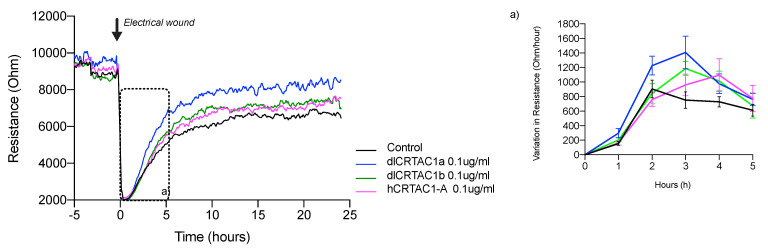
Electrical resistance of the confluent SJD.1 cell monolayer after scratching with an electrical discharge. A representative trace of the recovery of cell resistance after an electrical wound/ scratch (indicated by the arrow) is presented at 4 kHz. Changes in resistance are presented in different colors for each group of treated cells: control cells (black); hCRTAC1-A (pink), dlCrtac1a (blue) and dlCrtac1b (green). For each experimental group, the variation in resistance during the exponential phase of cell recovery (between 0 h–5 h after the wound/scratch) was estimated and is presented in insert (**a**). Data are presented as the average resistance of at least three independent experiments performed with two / three replicates for each experimental group.

The migration behavior of SJD.1 primary fibroblasts in the control medium or medium containing CRTAC1 proteins was determined using an ECIS system and recording resistance in the multiple frequency mode. For simplicity, the results were only presented at a frequency of 4 kHz for all experiments. At the start of the experiments microscopy was used to confirm that the electrode was covered with cells (data not shown) and the resistance measurements were high and had attained a stable plateau (Figure 2). At the start of the experiments the SJD.1 primary fibroblasts were detached from the sensing electrode surface by an electrical discharge. The creation of a wound/scratch by applying an optimized electrical discharge was visible as a drop in resistance to the levels measured with an empty electrode. The efficiency of wounding/scratching by an electrical discharge was confirmed by microscopy (Figure S3). Scratch recovery was monitored by measuring the change in resistance over time and occurred approximately 12 h post scratch and was evident as a stable plateau with similar values of resistance to those of the confluent cell layer before scratching (Figure 2).

The inclusion of CRTAC1 proteins (either hCRTAC1-A, 0.1 μg/mL, dlCrtac1a, 0.1 μg/mL or dlCrtac1b, 0.1 μg/mL) in the culture medium during the scratch repair caused a faster and higher increase in the resistance of the treated cells compared to the control cells. The presence of dlCrtac1a in the culture medium of electrically wounded SJD.1 primary fibroblasts caused a significantly (*p* < 0.05) faster recovery and a higher steady state resistance than the control (Figure 2). The scratch recovery rates were also estimated using regression analysis of the slope obtained from time 0 to the end of the exponential increase in resistance (time ± 5 h). Control SJD.1 primary fibroblasts had the lowest slope value (663) and had the slowest scratch recovery rate compared to the CRTAC1 treated cells. Cells treated with dlCrtac1a had the highest slope value (986) (fastest recovery), followed by dlCrtac1b (822) and hCRTAC1-A (803).

### 2.4. Cell Cytoskeleton Structures during Scratch Closure

**Figure 3:**
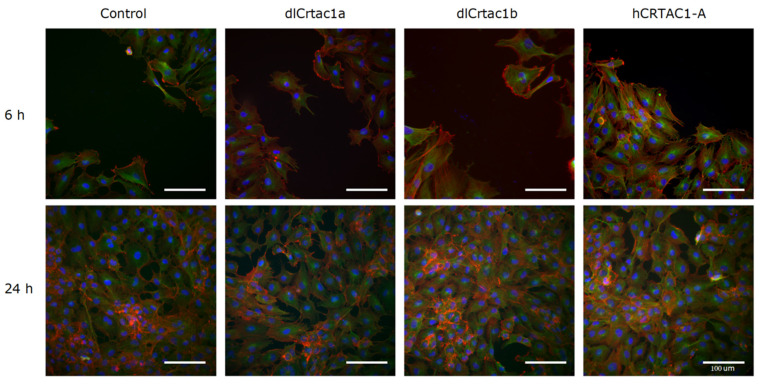
IF of SJD.1 after creating scratches in the confluent cells and treating them with CRTAC1 proteins (dlCrtac1a, dlCrtac1b and hCRTAC1-A) compared with the control cells. Colocalization of *α*-tubulin (green-fluorescence), F-actin (red fluorescence) and DAPI (nuclei, blue fluorescence). Images are representative of control cells, and CRTAC1 treated cells at 6 h and 24 h after scratching the confluent cell layer. Images were obtained with a Leica DM IL microscope coupled to a Visicam PRO 20C digital camera and photographs were analyzed using ImageJ software for image overlay. Scale bars indicate 100 μm.

Cell structure and shape are established by the actin cytoskeleton, the microtubule network and the intermediate filaments that together coordinate changes and promote cell migration [40,41]. To assess the changes that occurred in the cell cytoskeleton during scratch recovery IF of SJD.1 primary fibroblasts was performed 6 and 24 h after scratching, when the electrical cell resistance (ECIS) increased (Figure 2). Our IFA was focused on actin filaments and microtubules and revealed that the same general changes were observed in all CRTAC1 treatment groups. However, in more detailed analysis, it was observed that, CRTAC1 treated cells compared to control cells had an enhanced actin fluorescence, which was already visible at 6 h (Figure 3). When SJD.1 cells were actively migrating (cells at the edge of the scratch), 6 h after scratching, there was an enriched actin region in cells protruding from the edge of the scratch area. This actin-rich area consisted of a dense actin mesh forming ruffled lamellipodia containing actin bundles organized in filopodia (Figure 3, red fluorescence) and several actin-rich cell protrusions could be observed. In the center of the migrating cells at the edge of the scratch area, IFA revealed an enriched microtubule region spreading out from the cell nucleus and forming a fine network in the cytoplasm and toward the cell extremities (Figure 3, green fluorescence). At 24 h after scratching the SJD.1 cells behind the scratch edge were evenly spread and formed a confluent layer and at the scratch edge, the cells were more compact and contained actin rich regions in their cytoplasm (Figure 3). Additionally, qualitative observations suggested that dlCRTAC1b and hCRTAC1-A treated cells were more compact and smaller than the control and dlCRTAC1a treated cells, which appeared larger and with more empty spaces between cells.

### 2.5. Gene Expression of Genes Associated with Wound Healing

#### 2.5.1. Effect of Vertebrate CRTAC1 on Scratch Recovery/Cell Migration

**Figure 4:**
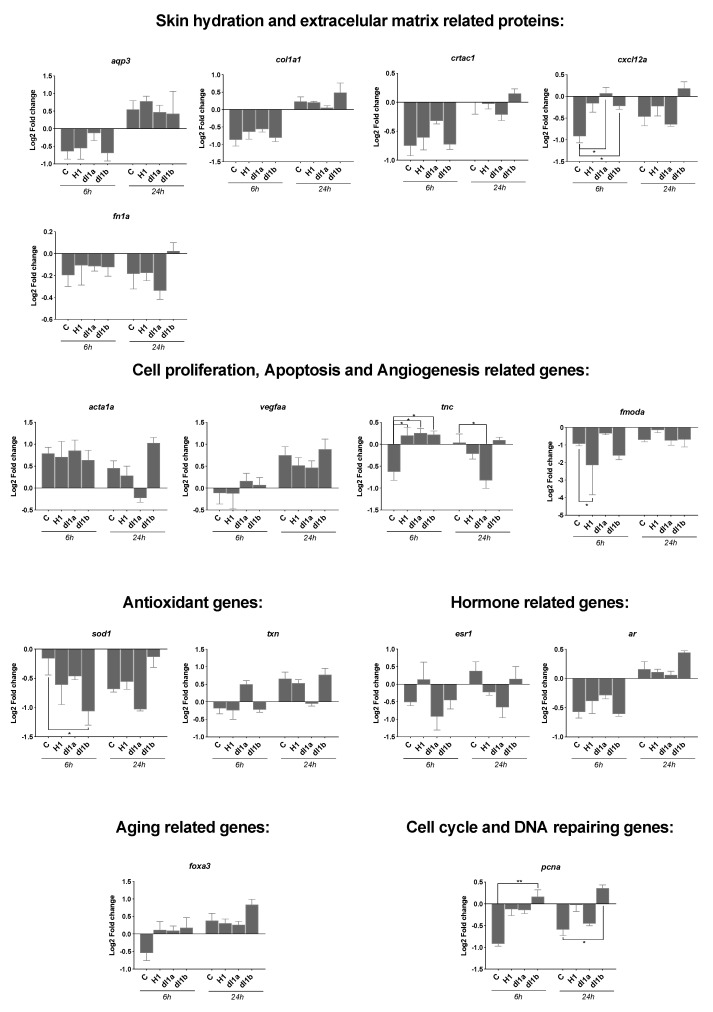
Transcript level of 15 genes quantified by RT-qPCR in SJD.1 fibroblast cells exposed to three control and CRTAC1 proteins (hCRTAC1-A, dlcrtac1a and dlcrtac1b). In intact cells (control) no damage was inflicted and other samples were collected immediately after the scratch (AS_0h) or at 6 h (AS_6h) and 24 h (AS_24h) after the scratch. Data corresponded to the mean ± SEM of four independent experiments for the control and dlCrtac1a and three independent experiments for hCRTAC1-A and dlCrtac1b. Gene expression levels (2^−ΔΔCT^) were normalized in relation to intact cells using the geometric mean of two reference genes (18S and ef1α) and the data were analyzed using a two-way ANOVA followed by Tukey’s Multiple Comparison test. The statistical analysis was performed using GraphPad Prism version 7.0a. *p* < 0.05 (*) and *p* < 0.01 (**) were considered significant. Gene symbols are indicated, for the full gene name consult Table 1.

The effect of hCRTAC1-A (0.1 μg/mL), dlCrtac1a (0.1 μg/mL) or dlCrtac1b (0.1 μg/mL) on gene expression by SJD.1 primary fibroblasts during scratch recovery was measured in cells before (control cells), immediately after (AS_0 h) and 6 and 24 h after scratching (AS_6 h and AS_24 h, respectively) (Figure 4). Analysis of genes associated with extracellular matrix related processes (*aqp3, col1a1a, crtac1a, cxcl12a* and *fn1*) revealed that the presence of dlCrtac1a and dlCrtac1b caused a significant increase (*p* < 0.05) in *cxcl12a* transcripts 6 h after scratching compared to the control.

Some transcripts associated with cell proliferation, apoptosis and angiogenesis, *acta1a* and *vegfaa*, were unchanged in SJD.1 primary fibroblasts exposed to hCRTAC1-A (0.1 μg/mL), dlCrtac1a (0.1 μg/mL) or dlCrtac1b (0.1 μg/mL) after scratching. However, the expression levels of other markers linked to the same process (*tnc* and *fmoda*) were changed in zebrafish SJD.1 primary fibroblasts exposed to hCRTAC1-A (0.1 μg/mL), dlCrtac1a (0.1 μg/mL) or dlCrtac1b (0.1 μg/mL). A significant up-regulation of *tnc* occurred at 6 h (*p* < 0.05) in cells exposed to hCRTAC1-A, dlCrtac1a or dlCrtac1b. A significant down-regulation occurred 24 h (*p* < 0.05) after scratching in SJD.1 cells treated with dlCrtac1a compared to control cells at the same time points. Exposure to hCRTAC1-A caused a significant down-regulation (*p* < 0.05) of *fmoda* 6 h after scratching compared to the control (Figure 4).

Gene transcripts associated with antioxidative activities (*sod1* and *txn*), the endocrine system (*esr1* and *ar1*) and cell development and differentiation (*foxa3*) were not significantly changed in SJD.1 primary fibroblasts exposed to vertebrate CRTAC1. The exception was *sod1* transcripts that were significantly decreased by dlCrtac1b 6 h after scratching compared to the control. The expression of transcripts for proliferating cell nuclear antigen (*pcna*) was significantly up-regulated (*p* < 0.05) 6 h and 24 h after the scratch in zebrafish SJD.1 primary fibroblasts exposed to dlCrtac1b compared to control cells (Figure 4).

#### 2.5.2. Effect of Time on Gene Expression during Scratch Recovery/Cell Migration

To assess the effect of time on gene expression during scratch recovery the expression levels of the target transcripts were compared immediately (AS_0 h), at 6 h (AS_6 h) and 24 h (AS_24 h) after the scratch within each treatment (Table 2). The transcript abundance of *fn1a*, *txn* and *esr1* did not change significantly across time in any of the treatment groups.

0 h vs. 6 h: None of the tested genes had a changed expression 6 h after scratching in the dlCrtac1a exposure group (Table 2). In the control cells 6 h after scratching *col1a1a* was significantly downregulated. Exposure of the SJD.1 primary fibroblasts to hCRTAC1-A caused a significant up-regulation of *acta1a* and a significant down regulation of *fmoda* 6 h after scratching. Significant down-regulated of *col1a1a*, *fmoda* and *sod1* and significant up-regulation of *pcna* occurred 6 h after scratching in SJD.1 primary fibroblasts exposed to dlCrtac1b compared to the control (Table 2).

0 h vs. 24 h: After 24 h exposure of zebrafish SJD.1 primary fibroblasts to hCRTAC1-A (0.1 μg/mL), dlCrtac1a (0.1 μg/mL) or dlCrtac1b (0.1 μg/mL) a greater number of genes had a significantly changed expression compared to the control immediately after the scratch (AS_0h) (Table 2). Exposure to hCRTAC1-A had a less significant effect on gene transcription and only *foxa3* was modified and significantly upregulated (*p* < 0.05). Three genes (*col1a1a*, *foxa3* and *sod1*) were significantly modified in the control and in the dlCrtac1a treatments (*p* < 0.05 and *p* < 0.01, respectively). Treatment of SJD.1 cells with dlCrtac1b for 24 h had the greatest effect on gene transcription and six genes, *cxcl12a*, *crtac1a*, *vegfaa*, *ar*, *foxa3* and *pcna* were significantly up-regulated (*p* < 0.05, *p* < 0.01 and *p* < 0.001) (Table 2).

6 h vs. 24 h: Of all the treatments analyzed the comparison of gene expression levels between 6 h and 24 h after scratching showed the highest number of genes with a significant change (Table 2). The majority of genes with different expression levels were upregulated 24 h after scratching. In the control group five genes (*col1a1a*, *crtac1a*, *vegfaa*, *ar* and *foxa3*), in hCRTAC1-A treatment four genes (*aqp3*, *col1a1a*, *acta1a* and *fmoda*) and in the dlCrtac1b three genes (*col1a1a*, *sod1* and *ar*) were significantly upregulated 24 h after inflicting a scratch on the confluent cell layer. In the dlCrtac1a treated SJD.1 primary fibroblasts there were only two genes (*tnc* and *acta1a*) with a significant change in their expression and both were downregulated 24 h after the scratch.

## 3. Discussion

The present study demonstrated that zebrafish SJD.1 primary fibroblasts are a good in vitro epidermal model for studies of their unique characteristics during damage repair. The SJD.1 cells were easy to maintain in culture, had a relatively quick doubling time and were responsive to treatments. The three forms of the tested ECM protein, CRTAC1 (hCRTAC1-A, dlCrtac1a and dlCrtac1b) enhanced zebrafish SJD.1 primary fibroblast scratch repair, although not in an identical manner. The rate of migration and repair was fastest for cells treated with hCRTAC1-A in the classical scratch assays and for cells treated with dlCrtac1a in the ECIS assays. Overall, the results indicated that CRTAC1 promoted cell migration and scratch repair by stimulating gene transcription and modifying the cytoskeleton to modulate the rate of cell migration. 

Classical scratch assays exploit the capacity of skin fibroblasts to rapidly repair damage. These assays mimic cell migration and wound healing in vivo [42,43]. A disadvantage of these assays is the high variability of the results and to overcome this issue we used an ECIS assay, which is more sensitive and based on the electrical impedance values of adherent cells. In our experiments, the results of the two approaches gave slightly different outcomes probably due to technical differences between assays such as, scratch type, mechanical (classical scratch assay) vs. electrical (ECIS assay), scratch size, which was much larger in the classical scratch assay (1 mm) compared to the ECIS assay (250 μM), and the scratch shape (linear vs. circular). The classical scratch assay may change the adherence and migration around the scratch area delaying cell migration something that does not occur in the ECIS assay. Furthermore, in the classical scratch assay cell clusters can form on the scratch edge and this does not occur in ECIS assay. These differences were reflected in the time of scratch repair, 24 h in the conventional assay and 8–12 h in the ECIS assay. Nonetheless, in both assays CRTAC1, irrespective of source (human or fish), enhanced migration and scratch recovery in SJD.1 primary fish fibroblast cells as was previously reported when human dermal fibroblasts were exposed to hCRTAC1-A [23]. The relatively well conserved sequence of CRTAC1 proteins between human and fish [22] presumably explain their similar capacity to enhance scratch repair in the zebrafish (SJD.1) primary fibroblasts. The differences in gene expression and the speed of scratch repair when hCRTAC1-A, dlCrtac1a or dlCrtac1b were applied to SJD.1 primary fibroblasts, may be explained by structural differences in the proteins. For example, hCRTAC1-A has a more stable structural conformation and a lower aggregation propensity than the fish proteins (dlCrtac1a and dlCrtac1b) [22,44] and dlCrtac1a is structurally more like hCRTAC1-A since both have a C-terminal calcium binding epidermal growth factor domain (EGF-Ca), which is missing in dlCrtac1b.

The action of CRTAC1 on promoting cell migration, which is reflected by the change in the cell cytoskeleton and gene transcription is coherent with the acknowledged importance of ECM proteins for structure, flexibility, organization, regulation of cell activity and function during wound repair [45]. Changes in the cell cytology of the SJD.1 primary fibroblasts treated with or without CRTAC1 was characteristic of migrating cells with clear cellular polarity and an organized microtubule network [40,41]. The zebrafish SJD.1 primary fibroblasts orientated themselves at the scratch edge and actin at the leading edge of the cells was organized into filopodia and a dense network formed lamellipodia, which has been associated with forward movement [40,41]. The involvement of actin in cell protrusion, contraction, and retraction movements has previously been described in human dermal fibroblasts [23,40,41] and the zebrafish SJD.1 primary fibroblasts had the same general characteristics. The addition of CRTAC1 promoted SJD.1 migration without disrupting the general characteristic of the cellular processes and cytoskeletal actin was much more abundant 6 h after scratching indicating that the effect of hCRTAC1-A, dlCrtac1a and dlCrtac1b on the cell cytoskeleton was rapid. The improved scratch repair response elicited by CRTAC1 in Normal Human Dermal Fibroblasts (NHDF) [23] and SJD.1 primary fibroblasts and the involvement of cytoskeletal actin is in line with the role of F-actin on cell shape [46,47,48] and migration [49].

To gain insight into the molecular basis of CRTAC1 effects on scratch repair by SJD.1 primary fibroblasts and to assess if the underpinning mechanisms were conserved in fish and human cells, gene transcripts related to the ECM, cell proliferation, cell migration and apoptosis were characterized. The transcriptional response associated with the effects of CRTAC1 on zebrafish SJD.1 primary fibroblasts and NHDF cell migration in a scratch assay differed [23]. Even considering the differences in the time course of the experiments with NHDF cells (24 h and 48 h) and SJD.1 fibroblasts (6 h and 24 h), the transcriptional response (*cxcl12a, fmoda*, *fn1* and *col1a1a*) of SJD.1 fibroblast cells was notably quicker and occurred during the early stage (6 h) of scratch repair. This difference is perhaps unsurprising when the characteristics of skin in fish and humans is considered along with their differing physiologies and the demands of their environments. Of the genes associated with extracellular matrix processes, the transcriptional response of chemokine, *cxcl12a*, involved in the inflammatory response [50,51] and fibroblast proliferation during wound healing [52,53], was significantly upregulated by hCRTAC1-A, dlCrtac1a and 1b in SJD.1 fibroblasts at 6 h but not 24 h after damage. *Cxcl12a* has previously been shown to regulate the migration of several different zebrafish cell types [54,55,56]. In contrast, *cxcl12* was strongly downregulated 24 h after scratching in NHDF cells treated with hCRTAC1-A [23] and this reinforces the differences at a transcriptional level of scratch repair in zebrafish and human cells. Fibromodulin (*fmoda*, a.k.a. Keratan sulfate proteoglycan fibromodulin) a leucine rich repeat proteoglycan was downregulated in SJD.1 fibroblasts 6 h after the scratch and was similar to control levels at 24 h. This contrasts, with NHDF cells, where scratch repair is slower and fibromodulin was upregulated at 24 h. The role of fibromodulin in ECM collagen fibers assembly [57] and in uncoupling pro-migration/contraction cell signals probably explains its changed expression during fibroblast scratch repair [58]. 

Overall, the results of the present and previous studies indicate that CRTAC1 induced cell migration is conserved in fish and mammalian cells. The mechanism by which CRTAC1 bring about this effect remains to be established, but short-term cytoskeletal reorganization and longer-term changes in gene expression are involved.

## 4. Materials and Methods

### 4.1. Culture of Zebrafish SJD.1 Primary Fibroblast Cells

The primary cell line, SJD.1 (ATCC^®^ CRL-2296^TM^), used in the study was isolated from fibroblasts of the caudal fin of zebrafish (*D. rerio*), a highly used fish model. The cryopreserved cells were defrosted and then cultures initiated following the supplier’s instructions. Cultures were maintained in a humid 8% CO_2_ incubator (Heraeus, Hanau, Germany) at 28 °C.

### 4.2. Cell Growth Assay

To establish the growth characteristics of the SJD.1 primary fibroblasts, a confluent monolayer of SJD.1 fibroblasts was dissociated from 5 mL culture flasks (Sarstedt, Nümbrecht, Germany) by Tryple^TM^ Select (1×) (Gibco, Thermo Fisher Scientific, Waltham, USA) digestion and seeded into 24-well cell culture plates (20,000 cells/well) in complete medium. Cells were maintained under optimal conditions (8% CO_2_ at 28 °C) and every other day cells from duplicate wells were harvested and manually counted over 15 days using a haemocytometer. Growth curves of SJD.1 primary fibroblasts were obtained using the results from two independent assays carried out with two technical replicates and contributed to the timeline selected for the scratch assay.

### 4.3. Scratch Assay

To evaluate the effect of the different vertebrate CRTAC1 proteins on cell migration a classical scratch assay was performed. For this assay, SJD.1 primary fibroblasts in 5 ml culture flasks (Sarstedt, Nümbrecht, Germany) from passage 2–3 were used. The cells were maintained in DMEM complete medium until they reached 70–80% confluence, at which time they were harvested with Tryple^TM^ Select (1×) to detach them from the culture dish, which were then plated into 6-well tissue culture plates (3 × 10^5^ cells/well) and incubated at 28 °C (8% CO_2_) until they achieved 90–100% confluence. When a confluent monolayer was obtained (approximately 48 h after seeding) multiple scratches were made in the confluent cell layer in each well using a 200 μL micropipette tip so that approximately 50 % of the adherent cells were removed (Figure S4). After scratching, the cells were washed twice with phosphate buffered saline (PBS) (1×) to remove the detached cells. Scratch repair experiments (*n* = 5 for each treatment) were conducted by incubating the remaining cells after scratching with: (1) normal DMEM medium (control); (2) DMEM medium supplemented with 0.1 μg/mL of human recombinant CRTAC1 (hCRTAC1-A); (3) DMEM medium supplemented with 0.1 μg/mL of seabass recombinant Crtac1a (dlCrtac1a) and (4) DMEM medium supplemented with 0.1 μg/mL of seabass recombinant Crtac1b (dlCrtac1b). The recombinant proteins were prepared in house as previously described [22] and preliminary dose-response experiments were used to select the working concentration (data not shown).

### 4.4. Electric Cell-Substrate Impedance Sensing (ECIS) Assays

The ECIS assay was performed to evaluate the effect of vertebrate CRTAC1 proteins on cell electrical parameters during migration after creating a wound using an electrical discharge. For this, an Electric Cell–Substrate Impedance Sensing instrument (ECIS Z⦵, Applied Biophysics, NY, USA) was used to record multiple frequency impedance measurements ranging from 62.5 to 64,000 Hz (62.5, 125, 250, 500, 1000, 2000, 4000, 8000, 16,000, 32,000, 64,000) and scratch closure was monitored under control conditions (normal DMEM medium) and in the presence of hCRTAC1-A, dlCrtac1a and dlCrtac1b recombinant proteins. In this study spectroscopic impedance data are presented at low frequency (4 kHz) as this frequency reflects a combination of intercellular (establishment of cell – cell junctions) and subcellular (cell – substrate adhesion) alterations as well as cell motility [59]. For the scratch assay, 8W1E devices (IBIDI), specifically designed for cell migration assays with a single 250 μM circular electrode covered in a thin gold film were used (Figure S5).

Briefly, 8W1E devices were washed, incubated with complete medium and stabilized in the ECIS system before seeding the cells. After stabilization, SJD.1 primary fibroblasts maintained in complete medium were seeded (50,000 cells/chamber) and allowed to grow to confluence under normal conditions. When cells reached confluence (indicated by a stationary impedance signal) a previously optimized current pulse (1200 μA, 40 kHz, 10 s) generated by the ECIS microelectrodes was applied to the cell monolayer to create a circular wound (Figure S3). After wounding/scratching the measurement was paused, cell monolayers were washed with complete medium to remove dead cells and normal complete medium or medium supplemented with hCRTAC1-A (0.1 μg/mL), dlCrtac1a (0.1 μg/mL) or dlCrtac1b (0.1 μg/mL) was added to the experimental groups. The scratch recovery process was then continuously measured for 24 h. The same wounding/scratching parameters were used in all experimental groups. For each experimental group at least three independent experiments were performed with two/three replicates in each.

### 4.5. Cell Cytoskeleton Immunofluorescence Assay (IF)

IF assays targeting cytoskeletal filaments (actin and tubulin) involved in cell migration were performed using SJD.1 primary fibroblasts during cell migration to understand the link between CRTAC1 treatments and scratch recovery. Briefly, zebrafish SJD.1 primary fibroblasts were seeded onto glass coverslips in 24-well plates (Sarstedt, Nümbrecht, Germany) and allowed to grow overnight under control conditions. When the cell monolayer reached confluence (approximately 24 h after seeding, confirmed by microscopic observation), it was scratched with a sterile micropipette tip (200 μL) (time 0 h). Cells was scratched with a sterile micropipette tip (200 μL). Cells were washed with complete DMEM medium to remove detached cells and then complete DMEM (control) or DMEM supplemented with hCRTAC1-A (0.1 μg/mL), dlCrtac1a (0.1 μg/mL) or dlCrtac1b (0.1 μg/mL) was added to the cells, and were incubated under standard conditions and the recovery of the monolayer monitored. For each experimental group at least three independent IF experiments were performed.

For IF analyses tubulin and actin filaments were observed 6 h and 24 h after damage. For the IF procedure, at 6 h and 24 h cells were rinsed in 1 × PBS and fixed in 4 % paraformaldehyde (4% PFA) by incubation for 15 minutes at room temperature and then rinsed again in 1 × PBS. IF was carried out using 4% PFA fixed cells permeabilized for 15 minutes in 0.1% Triton™ X-100, blocked for 1 h at room temperature with a 1 × PBS, 3% BSA solution and then incubated with a mouse monoclonal anti-α-tubulin antibody (1:300) (Sigma-Aldrich, Taufkirchen, Germany) in 1 × PBS, 3% BSA for 1 h at room temperature. Specificity of the anti-α-tubulin antibody for zebrafish α-tubulin is well characterized. The primary antibody was detected by incubation with goat anti-mouse IgG (H + L) secondary antibody conjugated to Alexa Fluor 488 (1:1000) (Thermo Fisher Scientific, Waltham, USA) in 1 × PBS, 3% BSA for 1 h at room temperature in the dark. Actin filaments were stained for 20 min at room temperature in the dark with Texas Red^®^-X Phalloidin (1:40) (Thermo Fisher Scientific, Waltham, MA, USA) in 1 × PBS and the nuclei were stained for 5 min at room temperature in the dark with a solution of 40,6-diamidine-20-phenylindole dihydrochloride (DAPI, 300 nM) (Acros organics, Waltham, MA, USA). Stained cells were covered with glycerol mounting media and stored in the dark until black and white digital images were captured using a Leica DM IL microscope coupled to a Visicam PRO 20C digital camera. Photographs were analyzed using ImageJ software [60] for colour and image overlay.

### 4.6. RNA Extraction and cDNA Synthesis

Total RNA (tRNA) was extracted from the control and CRTAC1 challenged cells using a Nucleospin RNA Kit (Macherey-Nagel, Duren, Germany) according to the manufacturer’s instructions. Cells were collected directly into the kit lysis buffer supplemented with β-mercaptoethanol and homogenized manually by pipetting up and down. A DNase I digestion protocol was performed directly on the Nucleospin columns during RNA extraction to eliminate contamination with genomic DNA. Total RNA was eluted in 40 µL of MilliQ water and quantified using a Nanodrop (Nanoquant, TECAN, Männedorf, Switzerland). The integrity of the total RNA was checked by running samples on a 1% TAE agarose gel. All samples were stored at −80 °C until use.

For cDNA synthesis, 500 ng of DNAse treated tRNA was used and cDNA synthesis was performed using a PrimeScript-RT reagent kit (Takara Bio Inc, Kusatsu, Shiga, Japan), following the manufacturer’s instructions. The quality and quantity of the cDNA synthesis was tested by PCR amplification of the human ribosomal subunit 18S rRNA (Table 1) using the following cycles: 95 °C for 3 min, followed by 25 cycles of 95 °C for 20 s, 58 °C for 20 s, 72 °C for 20 s and a final extension step of 72 °C for 5 min. The amplified PCR products were visualized on a 2% TAE agarose gel.

### 4.7. Gene Expression by Quantitative PCR

Real-time quantitative PCR (RT-qPCR) analysis was used to evaluate the change in expression of 15 zebrafish genes before and during scratch repair in control cells and cells supplemented with CRTAC1. The genes analyzed in this study were homologues of human genes previously shown to be involved in skin healing, cell migration and extracellular matrix related processes. Specific zebrafish primers were designed for each gene (Table 1) and RT-qPCR reactions were performed in duplicate (<5% variation between replicates) using a CFX connect TM Real Time System (Bio-Rad Laboratories, Hercules, CA, USA) and KAPA SyberGreen Fast reagent (Kapa Biosystems Inc., Wilmington, DE, USA). The reactions were prepared in a final volume of 10 µL with 200 nM of specific primers, and 2 µL of template cDNA (dilution 1:5) in low volume 96 - well plates. For each primer pair the annealing temperature was optimized (see Table 1). Optimized cycling conditions were: 5 min at 95 °C, followed by 40 cycles of 95 °C for 5 s and the optimized annealing temperature for the primer pair for 15 s. A final melting curve was performed between 60 °C and 95 °C and produced a single-product dissociation curve for each gene. The relative expression of the target transcripts was calculated using the 2^−ΔΔCT^ method using as the reference the geometric mean of elongation factor 1-alpha (*ef1α*) and 18S ribosomal subunit (*18 S*). The expression of ef1α and 18 s did not vary significantly between cDNA samples from different treatments and time points (data not shown). Relative expression of the target transcripts was normalized in relation to the control (non-damaged cell cultures).

### 4.8. Statistics

Statistical analysis of scratch recovery data (*n* = 5) was performed using a two-way ANOVA test followed by a Tukey’s Multiple Comparison test. In the ECIS experiment, a linear regression analysis was performed during the exponential phase of cell migration (0–5 h) to calculate the migration slope for each treatment (*n* = 3). To evaluate the effect of the scratch on gene expression levels an unpaired t-test (two-tailed) was used to compare cells before and after the scratch (*n* = 4 for all treatment except hCRTAC1 and dlCrtac1b for which *n* = 3). To assess the effect of time within each group and the effect of treatments at the same timepoint, a simple effects within columns test was used. For both analysis Tukey’s multiple comparison test was used as a follow-up. To evaluate the effect of vertebrate CRTAC1 on gene expression levels during scratch recovery a two-way ANOVA was performed. To assess the effect of the different vertebrate proteins within the same time point a simple effects within columns test was used followed by Tukey’s multiple comparisons test and to assess the effect of time within the same treatment a simple effects within rows test was used followed by Sidak’s multiple comparisons test. All statistical analysis was performed using GraphPad Prism version 7.0a for MacOSX, GraphPad Software, La Jolla California USA, www.graphpad.comand (accessed in July 2020). *p* < 0.0001 (****), *p* < 0.0005 (***), *p* < 0.01 (**) and *p* < 0.05 (*) were considered significant.

## 5. Conclusions

In summary, we showed that vertebrate CRTAC1 enhanced scratch repair in zebrafish SJD.1 primary fibroblast cells and that the immediate effect was through cytoskeletal reorganization and actin-driven cell migration. Our results also revealed that in the first stages of scratch repair, the presence of vertebrate CRTAC1 altered the expression of genes associated with extracellular matrix remodeling processes, cell proliferation, cell migration and apoptosis in zebrafish SJD.1 primary fibroblasts. Together these results demonstrate that although the molecular mechanisms behind the stimulatory effect of CRTAC1 on cell migration are still unknown, the CRTAC1 proteins show great potential as a bioactive compound in dermatological treatments. Our results also indicate that zebrafish SJD.1 primary fibroblast cells can be considered as a relevant in vitro model for skin-related research.

## Figures and Tables

**Table 1:** List of primers used in this study. For each primer pair, the full gene name, the gene abbreviation and the abbreviation of the human analogue, the annealing temperature (°C), the efficiency of the reaction (%) and the linearity (R^2^) of the standard curve are indicated.

Name	Sequence (5′-3′)	Annealing Temperature (°C)	Eff. (%)	R^2^
Superoxide dismutase 1	GCCGTTTGTGTGCTTAAAGG CCTGGAGTAAGGCCAGTAAT	60	99.4	0.99
Thioredoxin	CCTGACTATTCTAATGTGGTC GCTTCTTCCCGTTCTTGTAG	60	93.2	0.99
Vascular endothelial growth factor Aa	AGCTGCTGGTAGACATCATC TTCGAGCGCCTCATCATTAC	62	104.9	1.00
Actin alpha1, skeletal muscle a	CCACGATGTACCCTGGTATT GCCGATCCAGACTGAGTATT	62	104.1	1.00
Tenascin C	CCTGGGACTGAATATGGAATG GAAGGTCTTTGGGAGGATCA	62	101.7	0.99
Forkhead box A3	AGTCCAATTCGGGCAAAG CGTTTCTATGGCAGGAAGAG	62	106.8	1.00
Chemokine (C-X-C motif) ligand 12a	CAACACAGTCCCACAGAGAA GGGTTAATGCACACCTCCTT	60	96.3	0.99
Proliferating cell nuclear antigen	GACTCCTCTCATGTGTCTCT GCGTCAGCATTGTCTTCA	62	90.1	0.99
Collagen, type I, alpha 1a	CCTCCCAGAACATTACATACC CCCTCTGCTCTGATCTCAAT	60	98.3	0.99
Fibronectin 1a	ACCTCAGGTGCCTCCTATAA AGCTCCTGGAACGCTATTTC	62	106.6	1.00
Fibromodulin a	ACCTTCGTCTCAACCACAATA TCAGCCCAACACCAATATCC	62	105.4	0.99
Aquaporin 3a	CTTCACAGCCAGGGATTATTG CTTTCTTATCTCGTGCCTCTC	60	98.7	0.99
Cartilage acidic protein 1a	CGGGAGCCACAATAACAGAT GAGCCCTGGTTGACCTTAAA	60	105.3	0.99
Estrogen receptor1	TACGCCTCTGGATATCATTAC TGGTCGCTGGACAAACATAG	60	93.2	0.99
Androgen receptor 1	CTCCTCCTGTTTAGCGTCAT GTTGGTCTTCCTGCCATAGT	60	93.3	0.99
Ribosomal protein S18	TGACGGAAGGGCACCACCAG AATCGCTCCACCAACTAAGAACGC	58	96.9	0.99
Elongation factor 1 alpha	GTCCGTTCTTAGAGATACCA GACACAGAGACTTCATCAAG	58	98.5	1.00

**Table 2:** Genes with significantly different expression during scratch recovery (immediately after scratch (0 h), 6 h and 24 h after scratch) within the different treatments (control (C), hCRTAC1-A (H1), dlCrtac1a (dl1a) and dlCrtac1b (dl1b)). The direction of the arrow denote if the gene was upregulated (↑) or down regulated (↓) and the number of asterisks (*) denotes the *p*-level of the difference. *p* < 0.05 (*), *p* < 0.01 (**), *p* < 0.001 (***) and *p* < 0.0001 (****) were considered significant. Gene expression levels (2^−ΔΔCT)^ were normalized in relation to intact cells using the geometric mean of two reference genes (*18S* and *ef1α*). Four independent experiments for the control and dlCrtac1a and three independent experiments for hCRTAC1-A and dlCrtac1b were used. Data were analyzed using a two-way ANOVA followed by Sidak’s Multiple Comparison test using GraphPad Prism version 7.0a. Gene symbols are indicated, for full gene names consult Table 1.

		0 h vs. 6 h				0 h vs. 24 h				6 h vs. 24 h			
	Treatment	C	H1	dl1a	dl1b	C	H1	dl1a	dl1b	C	H1	dl1a	dl1b
Gene													
*aqp3*		-	-	-	-	-	-	-	-	-	* ↑	-	-
*col1a1a*		*↓	-	-	* ↓	-	-	* ↑	-	**** ↑	** ↑	-	**** ↑
*cxcl12a*		-	-	-	-	-	-	-	* ↑	-	-	-	-
*crtac1a*		-	-	-	-	-	-	-	* ↑	* ↑	-	-	-
*fn1a*		-	-	-	-	-	-	-	-	-	-	-	-
*vegfaa*		-	-	-	-	* ↑	-	-	* ↑	* ↑	-	-	-
*tnc*		-	-	-	-	-	-	-	-	-	-	** ↓	-
*acta1a*		-	* ↑	-	-	-	-	-	-	-	* ↑	** ↓	-
*fmoda*		-	*** ↓	-	** ↓	-	-	-	-	-	**** ↑	-	-
*txn*		-	-	-	-	-	-	-	-	-	-	-	-
*sod1*		-	-	-	** ↓	* ↓	-	** ↓	-	-	-	-	* ↑
*esr1*		-	-	-	-	-	-	-	-	-	-	-	-
*ar*		-	-	-	-	-	-	-	** ↑	** ↑	-	-	**** ↑
*foxa3*		-	-	-	-	** ↑	* ↑	* ↑	*** ↑	** ↑	-	-	-
*pcna*		-	-	-	* ↑	-	-	-	** ↑	-	-	-	-

Treatments: C (normal DMEM medium without proteins); H1 (cells treated with hCRTAC1-A); dl1a (cells treated with dlCrtac1a); dl1b (cells treated with dlCrtac1b).

## Supplementary Materials

The following are available online at https://www.mdpi.com/article/10.3390/md19100541/s1, Figure S1: SJD.1 cells growth curve in DMEM normal medium counted every other day over 15 days using an haematocytometer under normal conditions (8% CO_2_, and 28 °C), Figure S2: Immunofluorescent photos of SJD.1 cells in normal conditions. SJD.1 cells were labelled with a monoclonal anti-*α*-tubulin antibody detected with a IgG Secondary antibody (*α*-tubulin in green (b)), stained with Texas Red^®^-X Phalloidin (actin in red (c)) - DAPI (nuclei in blue (a)) and all images merged (d). Images were obtained by a Leica DM IL microscope coupled to a Visicam PRO 20C digital camera and photographs were analyzed using ImageJ software for image overlay. Scale bars indicate 100 μm, Figure S3: Images showing the result of the optimized wounding/ scratching protocol. Images of a single 250 μM circular electrode before (confluent SJD.1 cell monolayer (left)) and after (empty electrode (right)) of the optimized current pulse (1200 μA, 40 kHz, 10 s) generated by the ECIS equipment, Figure S4: Schematic representation of the scratch performed in the SJD.1 fibroblast monolayers to achieved multiple scratches that corresponded approximately to a scratch involving 50% of the cells, Figure S5: Schematic representation of 8W1E devices (IBIDI) used for cell migration ECIS assays with a single 250 μM circular electrode covered in a thin gold film.
