# Supplementary material for: Cartilage Acidic Protein a Novel Therapeutic Factor to Improve Skin Damage Repair?
Source: Mar Drugs. 2021 Sep 25;19(10):541. doi: 10.3390/md19100541 (PMC8536980; doi:10.3390/md19100541)

Supplementary Figures

Figure S1 - SJD.1 cells growth curve in DMEM normal medium counted every other day over 15 days using an haematocytometer under normal conditions (8 % CO_2, and 28 ºC).

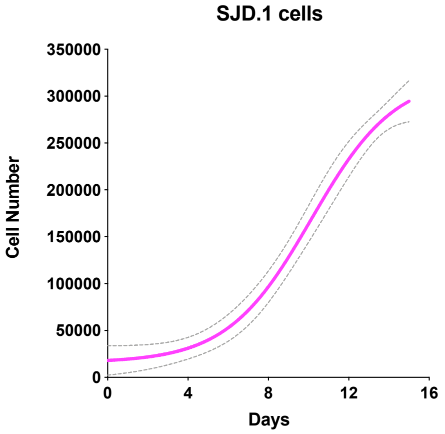

Figure S2 - Immunofluorescent photos of SJD.1 cells in normal conditions. SJD.1 cells were labelled with a monoclonal anti-α-tubulin antibody detected with a IgG Secondary antibody (α-tubulin in green (b)), stained with Texas Red®-X Phalloidin (actin in red (c)) and DAPI (nuclei in blue (a)) and all images merged (d). Images were obtained Leica DM IL microscope coupled to a Visicam PRO 20C digital camera and photographs were analyzed using ImageJ software for image overlay. Scale bars indicate 100 μm.

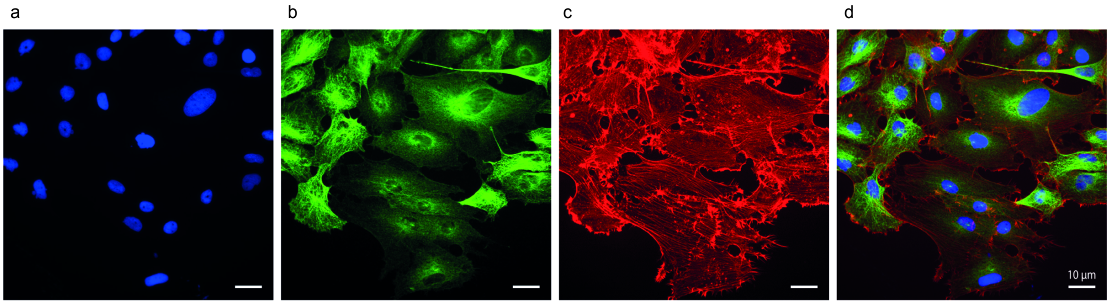

Figure S3 - Images showing the result of the optimized wounding/ scratching protocol. Images of a single 250 μM circular electrode before (confluent SJD.1 cell monolayer (left)) and after (empty electrode (right)) of the optimized current pulse (1200 μA, 40 kHz, 10 s) generated by the ECIS equipment.

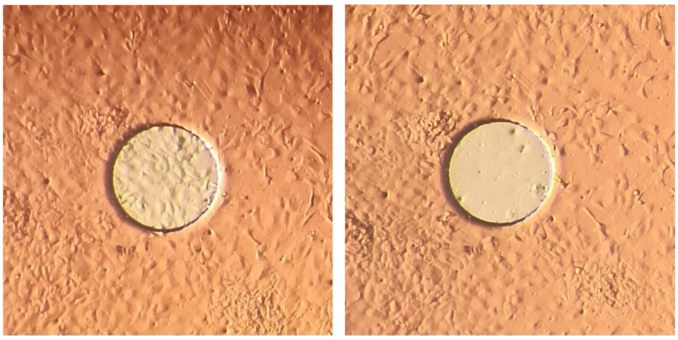

Before current pulse After current pulse

Figure S4 - Schematic representation of the scratch performed in the SJD.1 fibroblast monolayers to achieved multiple scratches that corresponded approximately to a scratch involving 50 % of the cells.

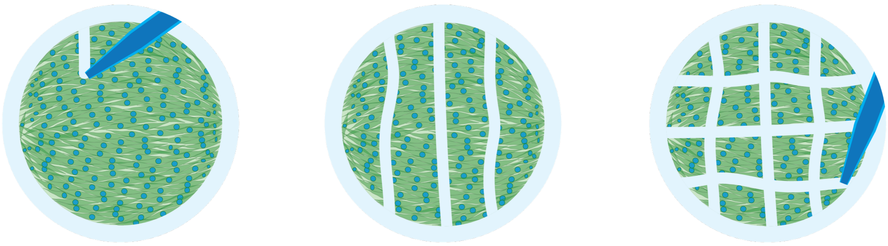

Figure S5 - Schematic representation of an 8W1E devices (IBIDI) used for cell migration ECIS assays with a single 250 μM circular electrode covered in a thin gold film.

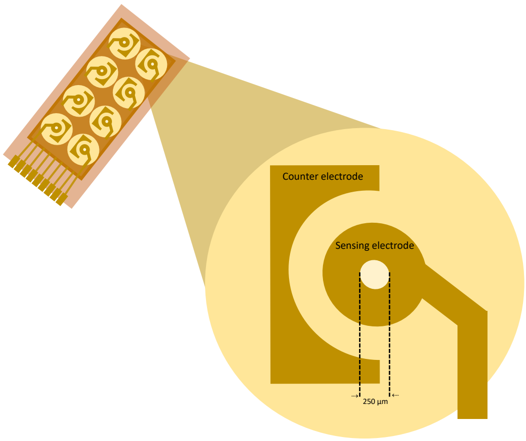

Supplement: Supplementary file 1 [file marinedrugs-19-00541-s001.zip › marinedrugs-1360640-supplementary.pdf]
